# Functional Diversity of AAA+ Protease Complexes in *Bacillus subtilis*

**DOI:** 10.3389/fmolb.2017.00044

**Published:** 2017-07-12

**Authors:** Alexander K. W. Elsholz, Marlene S. Birk, Emmanuelle Charpentier, Kürşad Turgay

**Affiliations:** ^1^Department of Regulation in Infection Biology, Max Planck Institute for Infection Biology Berlin, Germany; ^2^The Laboratory for Molecular Infection Sweden, Department of Molecular Biology, Umeå Centre for Microbial Research, Umeå University Umeå, Sweden; ^3^Humboldt University Berlin, Germany; ^4^Faculty of Natural Sciences, Institute of Microbiology, Leibniz Universität Hannover, Germany

**Keywords:** AAA+ protease complexes, Hsp100/Clp proteins, *Bacillus subtilis*, protein quality control, chaperones, regulatory proteolysis, McsB, adaptor proteins

## Abstract

Here, we review the diverse roles and functions of AAA+ protease complexes in protein homeostasis, control of stress response and cellular development pathways by regulatory and general proteolysis in the Gram-positive model organism *Bacillus subtilis*. We discuss in detail the intricate involvement of AAA+ protein complexes in controlling sporulation, the heat shock response and the role of adaptor proteins in these processes. The investigation of these protein complexes and their adaptor proteins has revealed their relevance for Gram-positive pathogens and their potential as targets for new antibiotics.

## Introduction

Bacteria, like all living organisms must rapidly sense and adapt to drastic changes in their environment (Roux, 1914). These environmental changes can directly or indirectly affect protein structure, activity and homeostasis. Protein quality control systems are an important part of cellular adjustment processes allowing a response to such changes. The conserved cellular protein quality control systems comprise chaperones and members of the AAA+ family, which can prevent or reverse the potentially toxic aggregation of misfolded proteins. Damaged, misfolded, or aggregated proteins that cannot be successfully refolded or repaired, can subsequently become degraded by the AAA+ protease complexes (Wickner et al., 1999; Hartl et al., 2011; Mogk et al., 2011).

These AAA+ proteins are members of a conserved family of ATP-hydrolyzing proteins with all kind of activities in many cellular pathways, including replication, DNA and protein transport, transcriptional regulation, ribosome biogenesis, membrane fusion, and protein disaggregation or degradation. The AAA+ family proteins often form hexamers, and can convert the energy of ATP hydrolysis into mechanical force in order to remodel or unfold proteins or nucleoprotein complexes, to move DNA or proteins, or to facilitate membrane fusion (Ogura and Wilkinson, 2001; Erzberger and Berger, 2006; Sauer and Baker, 2011).

**Figure 1:**
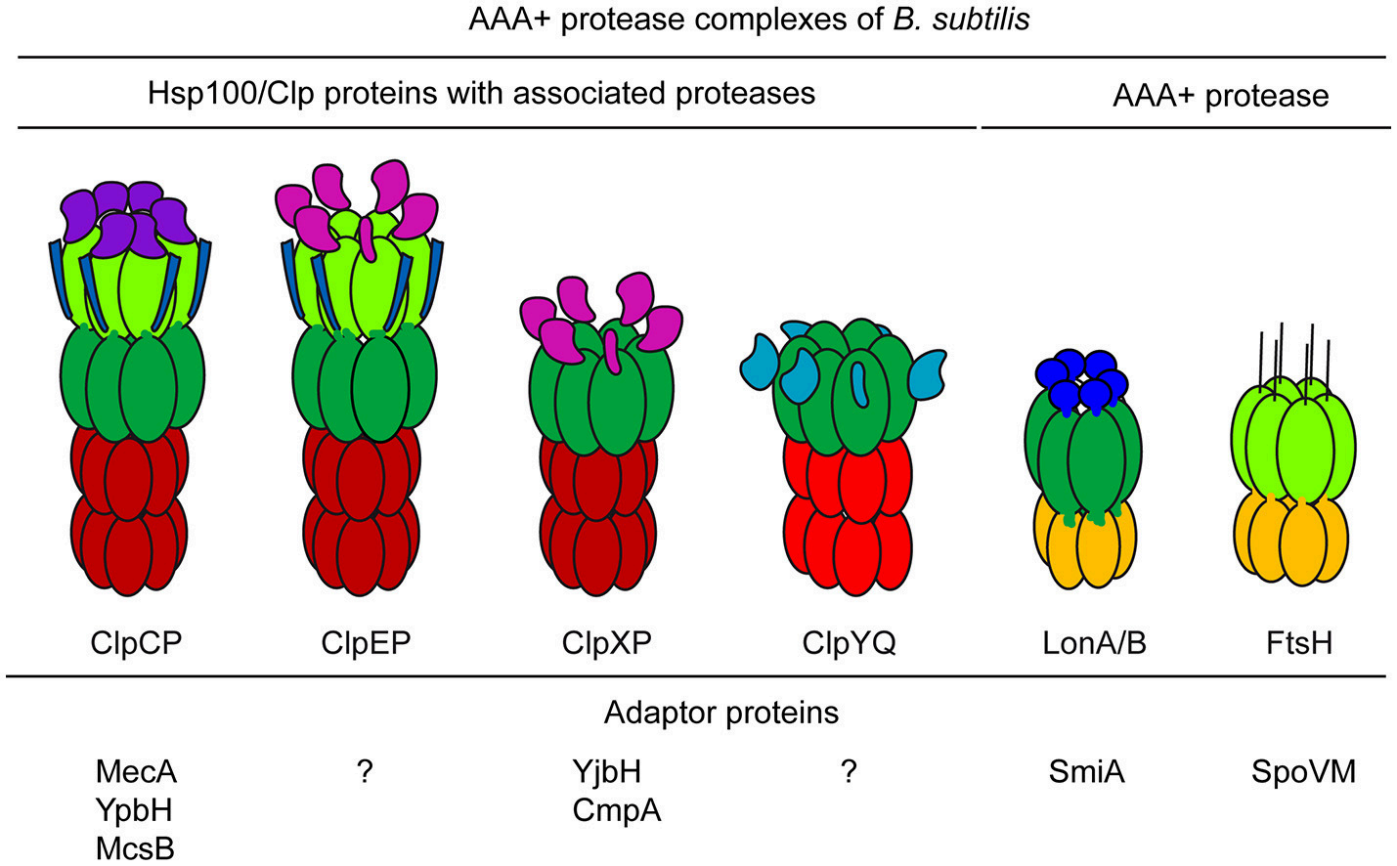
AAA+ proteases and adaptor proteins of *B. subtilis*. The AAA+ protease complexes of *B. subtilis* and the known interacting adaptor proteins are shown. The different distinguishing AAA+ and accessory domains are depicted.

The unifying activity of the AAA+ family proteins participating in protein quality control systems is to unfold proteins facilitated by ATP hydrolysis-dependent translocation using specific loops in the pore formed by the AAA+ hexameric ring structure. This unfoldase activity is central for the function of AAA+ proteins in protein disaggregation and degradation (Horwich et al., 1999; Sauer and Baker, 2011). In conjunction with Hsp70 chaperones, AAA+ proteins of the Hsp104/ClpB protein family can disaggregate and subsequently refold protein aggregates (Glover and Lindquist, 1998; Mogk et al., 2015). However, in AAA+ protease complexes, AAA+ unfoldases such as ClpC or ClpX associate with a specific barrel-shaped, compartmentalized protease complex, such as ClpP, which receive the unfolded proteins for degradation from the translocating AAA+ proteins (Weber-Ban et al., 1999; Wickner et al., 1999). Related AAA+ proteases such as Lon or FtsH form hexameric complexes, but encompass both, a AAA+ followed by a metallo-protease domain (Figure 1).

Interestingly, in the proteasome, the eukaryotic AAA+ protease complex, the base of the 19S regulatory subunit is consisting of AAA+ proteins forming a hetero-oligomeric hexamer, which is associated with the proteolytic 20S particle. Here, the heterologous AAA+ proteins play a similar role as homo-oligomeric hexameric AAA+ proteins in the bacterial AAA+ protease complexes of the Hsp100/Clp protein family (Kirstein et al., 2009b; Sauer and Baker, 2011; Matyskiela and Martin, 2013).

Specific sequence tags and/or adaptor proteins are necessary for the recognition, selection and preparation of substrate proteins for degradation by the AAA+ protease complexes. Diverse adaptor proteins for many AAA+ proteins have been characterized and identified in various bacteria, including model systems such as *Escherichia coli, B. subtilis*, or *Caulobacter crescentus*. The synthesis and activity of these adaptor proteins can be regulated by a variety of mechanisms and input signals. For example, adaptor protein activity can be controlled by sequestration, proteolysis, post-translational modification, or anti-adaptor proteins (Kirstein et al., 2009b; Sauer and Baker, 2011; Battesti and Gottesman, 2013; Joshi and Chien, 2016; Kuhlmann and Chien, 2017; Yeom et al., 2017). It was recently demonstrated in *E. coli* that DnaK selects and targets substrates for disaggregation and refolding by ClpB, and therefore can be considered an adaptor for ClpB (Weibezahn et al., 2004; Oguchi et al., 2012; Seyffer et al., 2012; Winkler et al., 2012b).

In *B. subtilis*, the ClpC adaptor proteins MecA, YpbH, and McsB, the ClpX adaptor proteins YjbH and CmpA, and the LonA adaptor protein SmiA were identified and characterized (Kirstein et al., 2009b; Mukherjee et al., 2015; Tan et al., 2015; Figure 1). Interestingly, the adaptor proteins of ClpC not only recognize substrate proteins, but also facilitate the activation of the ClpC hexamer, which allows for subsequent formation of the functional protease complex. In the absence of substrates, these adaptor proteins are themselves degraded, which leads to inactivation of ClpCP. This regulatory mechanism curbs the activity of the ClpCP protease when substrates are not present (Kirstein et al., 2006). In summary, adaptor proteins play an important role in controlling and facilitating the various and different regulatory and general functions of their cognate AAA+ proteins (Kirstein et al., 2009b; Sauer and Baker, 2011; Battesti and Gottesman, 2013; Joshi and Chien, 2016; Kuhlmann and Chien, 2017).

## Protein quality control and stress response systems in *Bacillus subtilis*

*B. subtilis* is considered the model organism for Gram-positive bacteria. *B. subtilis* cells are amenable to genetic manipulation, and many tools and methods exist for the study of its physiology and fundamental cellular processes (Sonenshein et al., 2002; Graumann, 2017). It is a soil-dwelling organism that can adjust to rapidly changing environmental conditions, including the availability of nutrients, water and oxygen, and changes in light, temperature, and salinity. This ability to sense and respond to various environmental stimuli is a prerequisite for the survival of *B. subtilis* in its ever-changing environment (Hecker and Völker, 2001). In addition to a number of general and specific stress response systems controlled by dedicated transcription factors (e.g., SigmaB, CtsR, HrcA, Spx, PerR, or OhrR; Hecker et al., 1996, 2007; Mogk et al., 1997; Zuber, 2009; Elsholz et al., 2010b; Runde et al., 2014), *B. subtilis* cells can also respond to environmental changes by triggering sophisticated and complex developmental programs that result in sporulation, biofilm formation, motility, or competence (Rudner and Losick, 2001; Errington, 2003; Chen et al., 2005; Lopez et al., 2009; Vlamakis et al., 2013; Mukherjee and Kearns, 2014; Hobley et al., 2015). The AAA+ protease systems and their adaptor proteins are intricately involved in stress response and developmental programs of *B. subtilis* cells. Consequently, pleiotropic effects were observed in *clpX, clpC*, and *clpP* deletion strains and these observed phenotypes are not only linked to protein quality control, but also imply a regulatory role for these genes in various stress response and developmental pathways (Dubnau and Roggiani, 1990; Msadek et al., 1994; Gerth et al., 1998; Kock et al., 2004; Zuber, 2004; Kirstein et al., 2009b; Runde et al., 2014).

### Role of AAA+ proteins and chaperone networks in *B. subtilis* protein homeostasis

The *B. subtilis* protein quality control system includes chaperones like the Hsp70 (DnaKJE) and Hsp60 (GroE) system, as well as other conserved chaperone systems such as ribosome-associated chaperones (Trigger factor), Hsp90 (HtpG), small heat shock proteins and redox chaperones (Schumann et al., 2002; Moliere and Turgay, 2009), together with AAA+ protease complexes.

The AAA+ unfoldase ClpB, which together with DnaK is necessary for protein refolding and disaggregation (Glover and Lindquist, 1998; Weibezahn et al., 2004; Haslberger et al., 2007; Winkler et al., 2010, 2012a; Oguchi et al., 2012; Seyffer et al., 2012), is widely conserved in most bacterial species, but is notably absent from *B. subtilis*. However, it was demonstrated that *B. subtilis* ClpC, which is closely related to ClpB, can—together with the adaptor protein MecA or its paralog YpbH—disaggregate and refold protein aggregates *in vitro* when not associated with ClpP (Schlothauer et al., 2003; Haslberger et al., 2008).

In *B. subtilis*, the AAA+ protease complexes ClpCP, ClpEP and ClpXP are part of the protein quality control system. ClpC was identified as a stress-induced protein, the Δ*clpC* strain is thermosensitive and, similar to Δ*clpP* or Δ*clpX* strains, display impaired degradation of misfolded proteins (Krüger et al., 1994, 2000; Msadek et al., 1994; Gerth et al., 1998, 2004; Kock et al., 2004). ClpE expression is tightly controlled and is only induced after severe heat shock, implying that ClpEP might function as an additional protease system under other severe stress conditions (Derre et al., 1999a; Gerth et al., 2004; Miethke et al., 2006). Consistent with their function in protein homeostasis, ClpC, ClpX, ClpE, and ClpP were all observed to associate with subcellular protein aggregates, especially upon heat shock or heterologous protein synthesis (Krüger et al., 2000; Jürgen et al., 2001; Miethke et al., 2006; Kain et al., 2008; Kirstein et al., 2008; Simmons et al., 2008).

As previously demonstrated for other bacteria (Sauer and Baker, 2011), ClpXP of *B. subtilis* is necessary for the degradation of proteins whose translation is stalled. These unfinished polypeptides are prone to aggregation and must be eliminated. In a process called trans-translation, stalled ribosomes are rescued by the activities of the SmpB protein in conjunction with the transfer and messenger RNA (tmRNA). The tmRNA is a specialized small RNA, which aided by SmpB first acts as a tRNA and subsequently like an mRNA. This not only liberates the ribosome, but also results in the addition of a short sequence, termed an SsrA tag to the C-terminus of the unfinished protein (Keiler et al., 1996; Muto et al., 2000; Abe et al., 2008; Keiler, 2008; Ujiie et al., 2009). ClpXP recognizes the C-terminal SsrA tag, and degrades these unfinished proteins, thereby preventing their aggregation (Keiler et al., 1996; Wiegert and Schumann, 2001; Sauer and Baker, 2011).

The membrane-associated FtsH AAA+ protease is most likely also directly involved in protein quality control, since a deletion of *ftsH* causes pleiotropic effects, including salt, and heat sensitivity (Deuerling et al., 1995, 1997). The two *B. subtilis* AAA+ protease Lon paralogs, LonA and LonB, do not have a significant role in the degradation of misfolded proteins (Riethdorf et al., 1994; Schmidt et al., 1994; Krüger et al., 2000; Serrano et al., 2001; Simmons et al., 2008). Only very little is known about the possible *in vivo* role of the *B. subtilis* ClpYQ (CodWX) AAA+ protease complex (Slack et al., 1995; Kang et al., 2003; Simmons et al., 2008; Figure 1).

### Role of chaperones and AAA+ protease complexes in controlling stress response pathways

An interesting feedback mechanism was observed for the regulation of chaperone synthesis in *B. subtilis*. The transcription of the *dnaK* and *groE* operon is controlled by the repressor HrcA, which is also encoded as the first gene of the *dnaK* operon. The GroEL chaperone is necessary for maintaining the repressor activity of HrcA. However, when GroEL interacts with unfolded proteins, HrcA repressor activity cannot be maintained and the synthesis of GroEL and DnaK is induced. The elevated levels of chaperones help to protect and repair the proteome. This subsequently restores the repressor activity of HrcA, thereby terminating the transcriptional induction of chaperones (Mogk et al., 1997; Schumann et al., 2002).

The same AAA+ protease complexes can be involved in general proteolysis for protein quality control and in regulatory proteolysis to control the activity of transcription factors and other key regulatory proteins. In *B. subtilis*, not only chaperones like GroEL are involved in sensing protein folding stress, but the AAA+ protease complexes ClpCP or ClpXP with their adaptor proteins McsB and YjbH are involved in sensing various stresses and are also involved in the regulation of their own synthesis by controlling e.g., CtsR or Spx stability (Zuber, 2004; Kirstein et al., 2009b; Rochat et al., 2012; Runde et al., 2014; Engman and von Wachenfeldt, 2015; Mijakovic et al., 2016).

#### Stress response and the control of the Spx regulon by ClpXP and its adaptor protein YjbH

**Figure 2:**
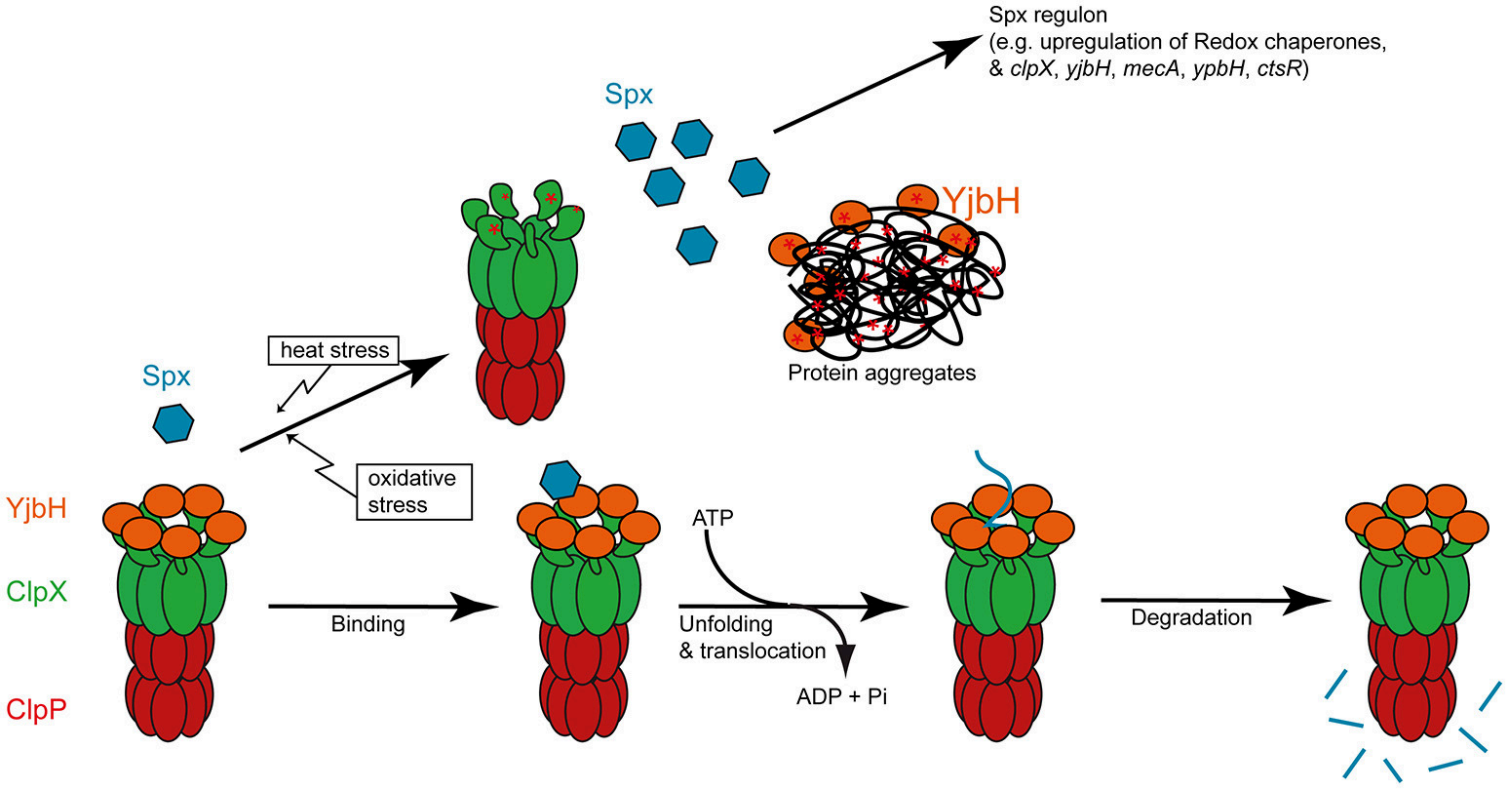
YjbH mediated degradation of Spx by ClpXP and its inhibition by heat and oxidative stress. The different steps of targeting of Spx by YjbH to ATP dependent degradation by ClpXP und non-stressed growth conditions is depicted in the lower part. Upon oxidative or heat stress, the adaptor protein YjbH is sequestered to subcellular protein aggregates (Engman and von Wachenfeldt, 2015). Both YjbH and the NTD can also become inactivated by oxidation (indicated by a ^*^ ; Zhang and Zuber, 2007; Garg et al., 2009).

The unusual transcription factor Spx was first identified by analyzing genetic suppressor mutations selected in a *clp*P or *clp*X deletion strain, which were mapped to the *yjbD* gene encoding Spx (Nakano et al., 2001). Spx is normally degraded by ClpXP, and the growth defect in *B. subtilis* strains lacking *clpX* or *clpP* is due to an accumulation of this transcription factor (Nakano et al., 2002, 2003a,b; Figure 2).

The same suppressor mutant analysis suggested, and subsequent structural analysis demonstrated, that Spx modulates transcription by interacting with the alpha subunit of the RNA polymerase (Nakano et al., 2000; Newberry et al., 2005). In doing so, it inhibits the interaction of activators with the RNA polymerase (Nakano et al., 2003b). In addition, it was observed that Spx can also operate at specific promoters as a redox-controlled activator of transcription (Nakano et al., 2005, 2010; Newberry et al., 2005; Lin and Zuber, 2012; Lin et al., 2013). Spx controls a broad regulon that includes genes important for the redox stress response, such as the redox chaperone TrxA and genes that maintain cellular thiol homeostasis (Antelmann et al., 2000; Nakano et al., 2003a,b; Zuber, 2009; Rochat et al., 2012). It was recently observed that not only oxidative stress but also heat stress can induce Spx activity and that Spx is essential for thermotolerance development in *B. subtilis*. These results suggested that Spx is important to orchestrate the heat and oxidative stress responses (Runde et al., 2014).

The stress sensing for the regulatory proteolysis and activity control of Spx is mediated via the adaptor protein YjbH and the N-terminal domain (NTD) of ClpX. Under normal conditions, ClpXP and the adaptor protein YjbH suppress Spx activity by mediating its degradation (Larsson et al., 2007; Rogstam et al., 2007; Garg et al., 2009; Kommineni et al., 2011; Chan et al., 2014). The adaptor protein YjbH induces the exposure of a ClpXP recognition element of Spx, thereby promoting Spx degradation under normal conditions (Chan et al., 2014). It was demonstrated that the zinc ion-containing NTD of ClpX is sensitive to oxidative stress, which would inhibit ClpXP mediated degradation. Spx activity can be directly modulated by disulfide bond formation upon oxidation of two specific cysteines (Nakano et al., 2005; Zhang and Zuber, 2007). Oxidative inactivation (Garg et al., 2009) or stress-mediated sequestration of YjbH to protein aggregates (Engman and von Wachenfeldt, 2015) results in the stabilization and accumulation of Spx also under heat stress conditions (Zuber, 2009; Runde et al., 2014). Therefore, multiple stress signals are sensed and integrated by the adaptor protein YjbH, the AAA+ protein ClpX and Spx itself in order to control the activity and stability of this transcription factor (Zuber, 2004, 2009; Figure 2).

Interestingly, a study combining global transcriptomics and identification of Spx chromosomal binding sites revealed that Spx activates not only transcription of the genes for the ClpC adaptor proteins MecA and YpbH (Nakano et al., 2003a), but also the genes for the AAA+ protein ClpX and its adaptor protein YjbH (Rochat et al., 2012). The same study provided evidence that Spx positively influences the expression of CtsR dependent genes. The observation of additional identified Spx binding sites might even suggest that HrcA-dependent gene expression could also be affected by Spx (Rochat et al., 2012). These results support a central and intricate role of Spx in *B. subtilis* heat shock response and protein quality control (Runde et al., 2014; Figure 2).

#### Heat and oxidative stress responses and the control of the CtsR regulon

CtsR (Class three stress repressor) is a global repressor of protein quality control genes in *B. subtilis* and all Gram positive bacteria (Elsholz et al., 2010a) and recognizes a conserved direct heptanucleotide repeat sequence in its dimeric form (Krüger and Hecker, 1998; Derre et al., 1999b). However, CtsR repressor activity is influenced by several different stress signals, and many of the signal transduction mechanisms that converge on CtsR are regulated by the protein quality control machinery (Elsholz et al., 2010a). Thus, CtsR represents a central regulator for the adaption of the cell to environmental changes that influence cellular protein quality control.

**Figure 3:**
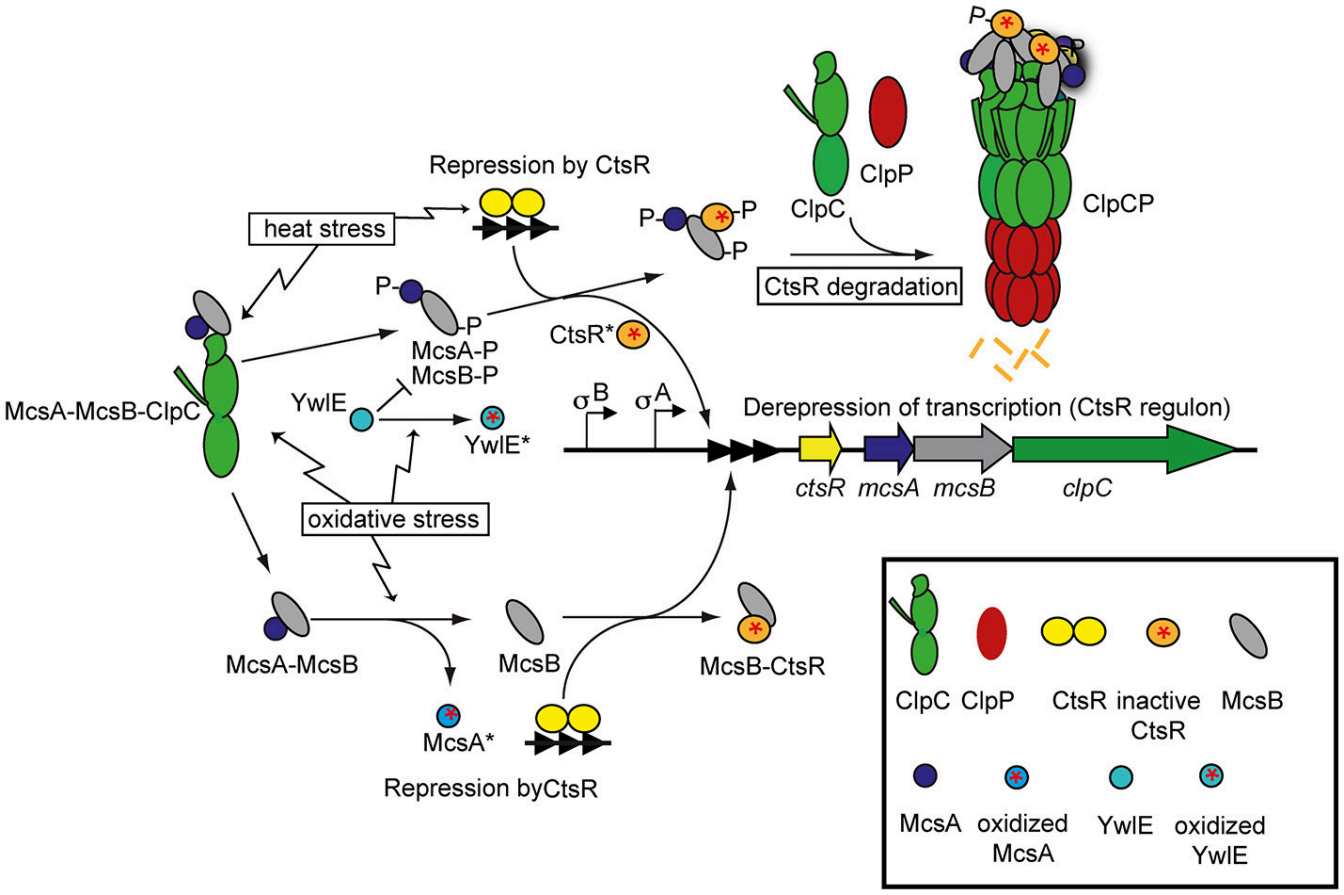
Regulation of CtsR activity under different environmental stress conditions. In non-stressed cells, CtsR is active as a repressor to inhibit expression of its target genes [class III heat shock genes (*clpP* and *clpE* not shown)] by binding to operator sites (three filled triangles). Upon heat exposure, CtsR is inactivated by an intrinsic thermosensor function of CtsR, resulting in the de-repression of its target genes. Free CtsR is targeted for ClpCP-dependent degradation by an active McsB kinase. Under normal conditions, McsB is kept inactive by interaction with ClpC. Heat stress results in the release and activation of McsB (which depends on the presence of the activator McsA). It is not known what factor results in the release of McsB, but we know that protein stress also leads to activation of the McsB kinase, which suggests that protein stress is responsible for the activation of McsB during heat stress. Once activated, McsB is not only able to target CtsR for ClpCP-dependent degradation, but also to phosphorylate CtsR, which further results in the inactivation of CtsR. Interestingly, during thiol-reactive stress conditions, McsA becomes oxidized. McsA not only acts as an activator of the McsB kinase, but also inhibits McsB-activity to directly remove CtsR from the DNA. However, oxidation of McsA^*^ disrupts its interaction with McsB, preventing McsB to act as a kinase but also allowing it to remove CtsR from the DNA, resulting in de-repression of the target genes. The protein arginine phosphatase YwlE, can dephosphorylate active McsB-P and thereby reset the McsB-P mediated inhibition of CtsR. However, YwlE is also prone to oxidation, and thereby its inhibitory effect can be relieved by oxidation (YwlE^*^).

CtsR controls the expression of its own operon containing *ctsR, mcsA, mcsB*, and *clpC*. *clpP* and *clpE* are also regulated by CtsR as single genes. CtsR therefore controls its own synthesis. *mcsA* and *mcsB* genes were identified as encoding modulators of CtsR activity (Krüger et al., 2001). Proteins like ClpC or ClpP whose expression is inhibited by CtsR play a crucial role for the adaptation to high temperatures and must be induced during heat stress in order to ensure survival of the cell (Krüger and Hecker, 1998; Derre et al., 1999b, 2000; Gerth et al., 2004). The level of control by CtsR is reflected by the number of CtsR binding sites in the respective promoters. The tighter the CtsR mediated repression is, the stronger the transcription of these proteins is repressed under optimal growth conditions and can be induced during stress conditions (Helmann et al., 2001; Petersohn et al., 2001). In contrast to what is known about the regulation of other heat stress response systems, the inactivation of CtsR during heat stress depends solely on an intrinsic thermosensing function, independent of other components such as chaperones influencing CtsR activity (Elsholz et al., 2010b; Figure 3). CtsR uses a highly conserved tetraglycine loop within the winged helix-turn-helix domain (HTH) to sense changes in temperature (Fuhrmann et al., 2009). This region possesses a high conformational entropy that confers decreased thermostability, and is conserved among all Gram-positive CtsR homologs (Elsholz et al., 2010b). Under non-stress conditions, CtsR binds to and represses its DNA operator. However, upon temperature upshift, the labile glycine-rich loop within the HTH changes conformation such that CtsR binding to DNA is impaired, and the expression of genes under the control of CtsR is induced. Interestingly, this ability of CtsR to sense changes in temperature is conserved among low-GC Gram-positive bacteria and adapted to the species-specific temperature of the ecological niche. This could suggest that the highly conserved tetraglycine loop is involved in the ability to sense temperature upshifts but that distinct, variable regions of CtsR are responsible for adaptation to species-specific temperatures (Elsholz et al., 2010a,b). Interestingly, CtsR-dependent gene expression becomes repressed upon heat exposure within 15 min (Elsholz et al., 2010b), showing that not the high temperatures itself, but rather the temperature upshift leads to CtsR inactivation. Newly synthesized CtsR molecules are able to bind to their DNA operators even under heat stress conditions, whereas inactivated CtsR molecules are targeted for ClpCP-dependent proteolysis.

##### ClpE-dependent control of CtsR activity

The mechanism described above allows expression of the CtsR regulon within minutes of exposure to heat (Krüger and Hecker, 1998). However, this CtsR mediated response is strictly limited in time, because newly synthesized active or reactivated CtsR can repress the transcription of its regulon again after about 15 min. Interestingly, the apparent reactivation of CtsR depends somehow also on the activity of the AAA+ protein ClpE. In a *clpE* mutant strain, CtsR is fully functional under normal growth temperatures and becomes inactivated upon heat exposure. However, the repression of CtsR-dependent gene expression is dramatically delayed in the absence of ClpE (Miethke et al., 2006). This observation indicates that ClpE—together with other AAA+ proteins such as ClpC—might be involved in maintaining the repressor activity of CtsR. This mechanism would ensure that expression of CtsR-regulated genes is only inhibited when appropriate levels of active AAA+ proteins are present to maintain CtsR activity (Miethke et al., 2006). How exactly the two diverging functions between CtsR-degradation and CtsR-reactivation are controlled and separated by the two AAA+ proteins, remains unclear, but for example an involvement through the control of McsB activity seems plausible. In a *clpE* or *clpC* mutant, the removal of protein stress conditions is delayed, which would keep the McsB kinase active for a longer time (Elsholz et al., 2011a), resulting in CtsR inactivation and thus delayed re-activation.

##### Regulation of CtsR by McsB

The most important regulator of CtsR is McsB, which is a protein arginine kinase and an adaptor protein for the ClpCP protease complex targeting specific substrates, such as CtsR, for ClpCP-dependent degradation. McsB is considered as a versatile protein that integrates different stress signals and fulfills a diverse set of functions (Kirstein et al., 2005, 2007; Fuhrmann et al., 2009, 2013; Elsholz et al., 2011b, 2012; Schmidt et al., 2014; Mijakovic et al., 2016).

##### McsB as a protein kinase and its control by ClpC, McsA and YwlE

Protein arginine phosphorylation by McsB can drastically change protein activity by switching the charge of the protein at the phosphorylation site and/or by targeting the protein for degradation (Kirstein et al., 2005, 2007; Fuhrmann et al., 2009; Elsholz et al., 2012; Trentini et al., 2016). Therefore, McsB kinase activity must be stringently controlled. Consistent with this, cells expressing hyperactive McsB display a severe growth defect (Elsholz et al., 2011a).

The activity of the McsB kinase is tightly controlled by a complex regulatory network that involves its activator McsA, the AAA+ proteins ClpC and ClpE, as well as the recently identified protein arginine phosphatase YwlE (Kirstein et al., 2005; Elsholz et al., 2011a; Mijakovic et al., 2016). Auto-phosphorylation of McsB is thought to promote its activation (Kirstein et al., 2005; Elsholz et al., 2011a, 2012; Fuhrmann et al., 2013). YwlE is the cognate phosphatase for McsB-dependent arginine phosphorylation events (Kirstein et al., 2005; Elsholz et al., 2011a, 2012; Fuhrmann et al., 2013) and YwlE counteracts McsB function not only by de-phosphorylating its substrates, but also by dephosphorylating McsB itself (Figure 3).

ClpC and ClpE both act as inhibitors of McsB activity (Kirstein et al., 2005, 2007; Elsholz et al., 2011a). It has been shown that the McsB kinase activity is strongly inhibited by ClpC *in vitro* (Kirstein et al., 2005) and that McsB strongly interacts with ClpC *in vivo* due to a translation coupling of McsB with ClpC, but that this interaction is abolished upon stress induction. Moreover, in the absence of ClpC, McsB kinase activity is observed even in the absence of any stress conditions (Elsholz et al., 2011a). These observations suggest that under non-stress conditions, McsB interacts with ClpC and that this interaction inhibits McsB activation. Upon stress induction, McsB is released from ClpC inhibition and is free to phosphorylate its target proteins. Interestingly, the release of McsB from ClpC activate McsB as a protein arginine kinase and adaptor protein (Kirstein et al., 2005, 2007; Elsholz et al., 2011a; Figure 3).

Interestingly, McsB not only promotes protein degradation, but also inhibits the repressor activity of CtsR, possibly by phosphorylating CtsR within the DNA-binding domain (Kirstein et al., 2005, 2007; Fuhrmann et al., 2009; Elsholz et al., 2011a). Although McsB is not involved in the inactivation of CtsR upon heat stress, it has been shown that McsB kinase activity results in CtsR inactivation *in vivo* (Elsholz et al., 2010b, 2011a). This regulatory mechanism might explain the inactivation of CtsR under other stress conditions that have been shown to strongly activate CtsR-dependent gene expression, including salt and protein folding stress. A common cellular event that is induced by all these different stress conditions is protein misfolding and aggregation, which could directly or indirectly affect this inhibitory interaction between ClpC and McsB (Kirstein et al., 2007; Elsholz et al., 2011a; Figure 3). The activation of McsB might represent a regulatory mechanism that monitors the level of protein stress in the cell and ties the protein homeostatic state of the cell to the expression and activity of protein quality control systems. In addition, McsB has been shown to phosphorylate hundreds of proteins including many regulatory proteins (Elsholz et al., 2012; Schmidt et al., 2014; Trentini et al., 2016). Thus, it is conceivable that McsB might influence a wide range of cellular processes.

##### Sensing of oxidative stress via McsA and YwlE

As mentioned above, McsB kinase activity is inhibited not only by the association with the AAA+ protein ClpC, but also by the protein arginine phosphatase YwlE (Elsholz et al., 2012; Schmidt et al., 2014; Figure 3). Although, YwlE shows a strong homology to low-molecular weight protein tyrosine phosphatase (LMWPTP), it de-phosphorylates arginine rather than tyrosine residues (Fuhrmann et al., 2013). This selectivity for phospho-arginine residues depends on a single amino acid change (Fuhrmann et al., 2013). Interestingly, the active center of LMWPTPs and YwlE contains a cysteine residue that is susceptible to oxidative damage (Chiarugi and Cirri, 2003; Fuhrmann et al., 2016). Recently, Fuhrmann and colleagues showed that YwlE is indeed subject to regulation through oxidation of this critical cysteine residue under certain oxidative stress conditions, such as exposure to H_2_O_2_ (Fuhrmann et al., 2016). Once this cysteine residue in the active center is oxidized, YwlE becomes inactive, resulting in the partial activation of the McsB kinase (Fuhrmann et al., 2016; Figure 3). This specific regulatory circuit involving YwlE illustrates another way by which oxidative stress promotes McsB-dependent regulation of diverse cellular processes.

Interestingly, these two molecular mechanisms are not the only regulatory circuits that influence the activity of CtsR and its associated protein quality control networks. It has been shown that CtsR is inactivated during thiol-reactive stress conditions. Under these stress conditions, CtsR inactivation depends on a redox-dependent partner switching mechanism involving McsA and McsB. Under normal growth conditions, McsA strongly interacts with McsB. This not only activates the McsB kinase, but also inhibits McsB binding to and inactivation of DNA-bound CtsR (Figure 3).

McsA is a redox-sensing protein whose activity depends on the redox state of its thiols. Oxidation of these thiols prevents interaction of McsA with McsB. Liberated McsB is no longer inhibited by McsA and is thus able to remove CtsR from the DNA (Elsholz et al., 2011b; Figure 3).

This molecular redox switch not only controls the expression of CtsR-dependent protein quality control systems, but also influences their activity directly. Interaction of McsB with McsA is required for its kinase activity, which is in turn necessary for the role of McsB as an adaptor that promotes protein degradation by ClpCP (Kirstein et al., 2007). During thiol-reactive stress, McsA oxidation not only promotes McsB-dependent removal of DNA-bound CtsR, but also prevents McsB kinase activity (Elsholz et al., 2011b), thus also influencing the activity of ClpC (Figure 3). Interestingly, in low GC Gram-positive bacteria that lack McsA and McsB, ClpE might be able to sense and respond to oxidative stress. The NTD of ClpE is homologous to the NTD of ClpX, which contains a Zn-binding site, known to render ClpX sensitive to oxidation (Zhang and Zuber, 2007; Garg et al., 2009). This suggests that the NTD of ClpE like the NTD of ClpX could act as a sensor for oxidative stress. Thereby ClpE could sense stress and induce the CtsR operon in these organisms, since the inactivated ClpE might not be able to activate CtsR any longer (Elsholz et al., 2011b).

### The general role of ClpC and McsB in cellular protein quality control

As mentioned above, McsB can act as an adaptor for the AAA+ protein ClpC. It has been shown that this activity depends on the ability of McsB to function as a protein kinase (Kirstein et al., 2005, 2007). Only when active as a kinase McsB can stimulate ClpC activity, and this specific activation depends on site-specific phosphorylation of ClpC by McsB (Elsholz et al., 2010b). The kinase activity of McsB has also shown to be required for the degradation of specific substrates by the ClpCP protease. However, McsB might be involved in regulatory proteolysis of not only transcription factors such as CtsR, but also other proteins. There are strong indications that the ClpC adaptor proteins McsB like MecA or YpbH play an important role together with ClpCP not only in regulatory proteolysis of CtsR, but also in general proteolysis and protein quality control (Kirstein et al., 2008).

#### McsB and protein quality control

Heat stress promotes the kinase activity of McsB and promotes the association of McsB with subcellular protein aggregates at the poles. ClpC and ClpX are also recruited to these aggregates but in an McsB-independent manner (Kirstein et al., 2008). Interestingly, in an *mcsB* deletion strain the misfolded protein, GudB^*^, accumulates at the cell pole (Stannek et al., 2014), where it probably associates with protein aggregates. This observation could suggest a possible scenario where McsB together with ClpC or ClpE is important to disassemble small protein aggregates prior to degradation or reactivation facilitated by the chaperone system. Moreover, McsB and ClpC have been implicated in the disassembly of the competence apparatus, which is also located at the poles. Here the accumulation of a component of the competence apparatus ComGA-GFP fusion gave the first indication of such a mechanism (Hahn et al., 2009). This suggests the possibility that McsB, like the other proteins encoded in the CtsR regulon, is also a central player of the protein quality control system.

##### Direct recognition of unfolded arginine-phosphorylated proteins by ClpCP

The arginine kinase activity of McsB is required for its ability to stimulate ClpC activity and to promote degradation of its substrates by the ClpCP protease. This makes it difficult to dissect the kinase and adaptor activities of phosphorylated McsB (Kirstein et al., 2007). Nevertheless, it was recently demonstrated that the NTD of ClpC can directly recognize phosphorylated arginines at two binding sites. An *in vitro* arginine-phosphorylated artificial protein substrate, the naturally unfolded beta-casein, could alone activate ClpC and was degraded by ClpCP without the presence of McsB and McsA (Trentini et al., 2016). These experiments demonstrate that ClpCP alone can recognize and degrade an arginine phosphorylated protein suggesting a new possible recognition tag for ClpCP-mediated protein degradation, and expanding the known repertoire of degradation tags for controlled protein degradation mechanism in bacteria (Trentini et al., 2016).

However, it should be noted that another ClpCP substrate, the arginine-phosphorylated CtsR, is not recognized and degraded by ClpCP in the absence of McsB and that CtsR phosphorylation on arginine residues is not sufficient for its targeting for degradation by ClpCP (Kirstein et al., 2007). It is possible that beta-caseine, which is an unfolded protein might itself be recognized directly by the NTD of ClpC (Erbse et al., 2008) in addition to the recognition of its phosphorylated arginines. Arginine-phosphorylated unfolded beta caseine might participate in activating ClpC and become targeted by degradation because of these two distinct interactions with ClpC. Nevertheless, these results suggest that during heat stress, McsB might phosphorylate unfolded or aggregated proteins to mark them for subsequent ClpCP degradation, however that might not apply to other proteins targeted by McsB for ClpCP degradation. A ClpC variant with mutations in both Arg-P binding sites (ClpC^EA^) did not complement a *clpC* deletion strain for survival during heat stress (Trentini et al., 2016), suggesting the possibility of a more general protein quality control role of protein arginine phosphorylation. However, it is not yet understood how McsB activates ClpC. Therefore, the complex interaction between McsB as adaptor and kinase, its substrate and the NTD of ClpC have to be sorted out before a more definitive understanding of the role of McsB as adaptor protein and arginine protein kinase during heat stress in *B. subtilis* cells can be reached. To fully understand the role of arginine phosphorylation, McsB, and ClpC in general protein quality control, further *in vivo* and *in vitro* studies should be conducted.

## AAA+ protease complexes and the control of regulatory and cell developmental pathways of *B. subtilis*

Regulatory proteolysis represents a very fast and efficient cellular control mechanism (Jenal and Hengge-Aronis, 2003). Therefore, it comes as no surprise that the *B. subtilis* AAA+ protease complexes are not only intricately involved in protein quality control and in sensing and responding to stress, but are also engaged in the initiation and control of distinct cellular developmental processes of *B. subtilis*.

In the ever-changing environment encountered by bacteria, the ability to differentiate into specialized cell types is a crucial survival strategy. Complex developmental processes are a hallmark of *B. subtilis* and AAA+ proteases play crucial roles for the regulation of these cellular processes.

### Competence

When grown into stationary phase, a subpopulation of *B. subtilis* cells develop the ability to actively take up extracellular DNA. ComK is the transcription factor necessary and sufficient to induce the transcription of the competence state (K-state) regulon. ComK induces the transcription of competence genes, which encode the proteins necessary to form the DNA receptors that recognize and transport extracellular DNA into the cell. Concurrently, DNA repair and recombination systems are upregulated, whereas general transcription, translation, cell division and growth are impaired (van Sinderen et al., 1995; Haijema et al., 2001; Berka et al., 2002; Hamoen et al., 2003; Chen et al., 2005; Hahn et al., 2005, 2015). Thus, the K-state cells are not only able to take up DNA, but also exhibit properties such as growth inhibition that are characteristic of persister-like cellular states (Hahn et al., 2015), and which can confer a survival advantage in the face of antibiotics or other stressors (Yüksel et al., 2016).

In exponentially growing *B. subtilis* cells, ComK is constantly antagonized by the adaptor protein MecA. MecA not only targets ComK for degradation by ClpCP, but also directly inhibits ComK activity (Dubnau and Roggiani, 1990; Kong and Dubnau, 1994; Turgay et al., 1997, 1998; Persuh et al., 1999). At higher cell density in post-exponential cells, signaling via a quorum sensing system causes the stable phosphorylation of the response regulator ComA, which results in the synthesis of the small protein ComS (D'Souza et al., 1994; Hamoen et al., 1995). ComS competes with ComK for binding to MecA (Prepiak and Dubnau, 2007), which results in the release of ComK from MecA-mediated inhibition and degradation (Turgay et al., 1997, 1998). Since ComK is a positive autoregulatory transcription factor, this release results in the exponential synthesis of ComK in the subpopulation of competence-developing *B. subtilis* cells. The MecA-dependent retargeting of the abundant ComK protein for ClpCP degradation is essential for the escape from competence (Turgay et al., 1998).

This post-translational regulatory mechanism—where the activity of an adaptor protein is controlled by the signal-induced synthesis of a small protein that acts like an anti-adaptor protein—was also observed in *E. coli* for the proteolytic control of the general stress sigma factor σ^S^ by the adaptor protein RssB-P (Becker et al., 1999; Bougdour et al., 2006; Hengge, 2009; Battesti and Gottesman, 2013; Battesti et al., 2013; Micevski et al., 2015).

### Sporulation

Endospore formation is a terminal cellular developmental process leading to two different types of cells in a structure termed the sporangium. This event begins with asymmetric cell division, after which the larger mother cell encloses the smaller forespore cell and supports its development into an endospore. This concerted cellular developmental process culminates in the release of the endospore from the lysing mother cell (Rudner and Losick, 2001; Higgins and Dworkin, 2012). The endospore is metabolically inactive and highly resistant to most stressors and environmental extremes (Piggot and Hilbert, 2004). Once the cell has committed to this developmental process, it is irreversible (Dworkin and Losick, 2005). Consequently, half of the progeny will transform into an endospore, whereas the other half will die. It is therefore critical that this process is tightly regulated. Indeed, the decision whether or not to commit to this complex developmental process is controlled by multiple regulatory circuits that integrate several distinct signals (Higgins and Dworkin, 2012). Interestingly, AAA+ protease complexes have several important roles at various stages of this complex decision-making process. The roles of ClpCP, ClpXP and FtsH sporulation have been elucidated in detail (Pan et al., 2001; Bradshaw and Losick, 2015; Tan et al., 2015).

One of the interesting aspects of sporulation is an asymmetric cell division that results in two unequally sized daughter cells a smaller forespore and a larger mother cell. Upon asymmetric division, both cells engage specific and distinct gene expression programs that ultimately determine their markedly different fates (Piggot and Hilbert, 2004). The first cell type-specific genetic program is the activation of the alternative sigma factor F in the forespore, which depends on both a partner-switching mechanism involving the anti-sigma factor SpoIIAB and the anti-anti-sigma factor SpoIIAA, and also on the activity of the PP2C phosphatase SpoIIE (Stragier and Losick, 1996).

Sigma F and all factors required for its activation are produced at the onset of sporulation and thus are present in both cell compartments (Gholamhoseinian and Piggot, 1989). For over two decades it was not understood how Sigma F is activated exclusively in the forespore. SpoIIE is the critical controller of the activation of Sigma F: it de-phosphorylates SpoIIAA, which can then activate Sigma F (Stragier and Losick, 1996). Intriguingly, SpoIIE is expressed in both compartments but the protein is found only in the forespore (Gholamhoseinian and Piggot, 1989). Bradshaw and Losick recently implicated the AAA+ protease FtsH in the compartment specific regulation of SpoIIE stability during the early stages of sporulation (Bradshaw and Losick, 2015).

**Figure 4:**
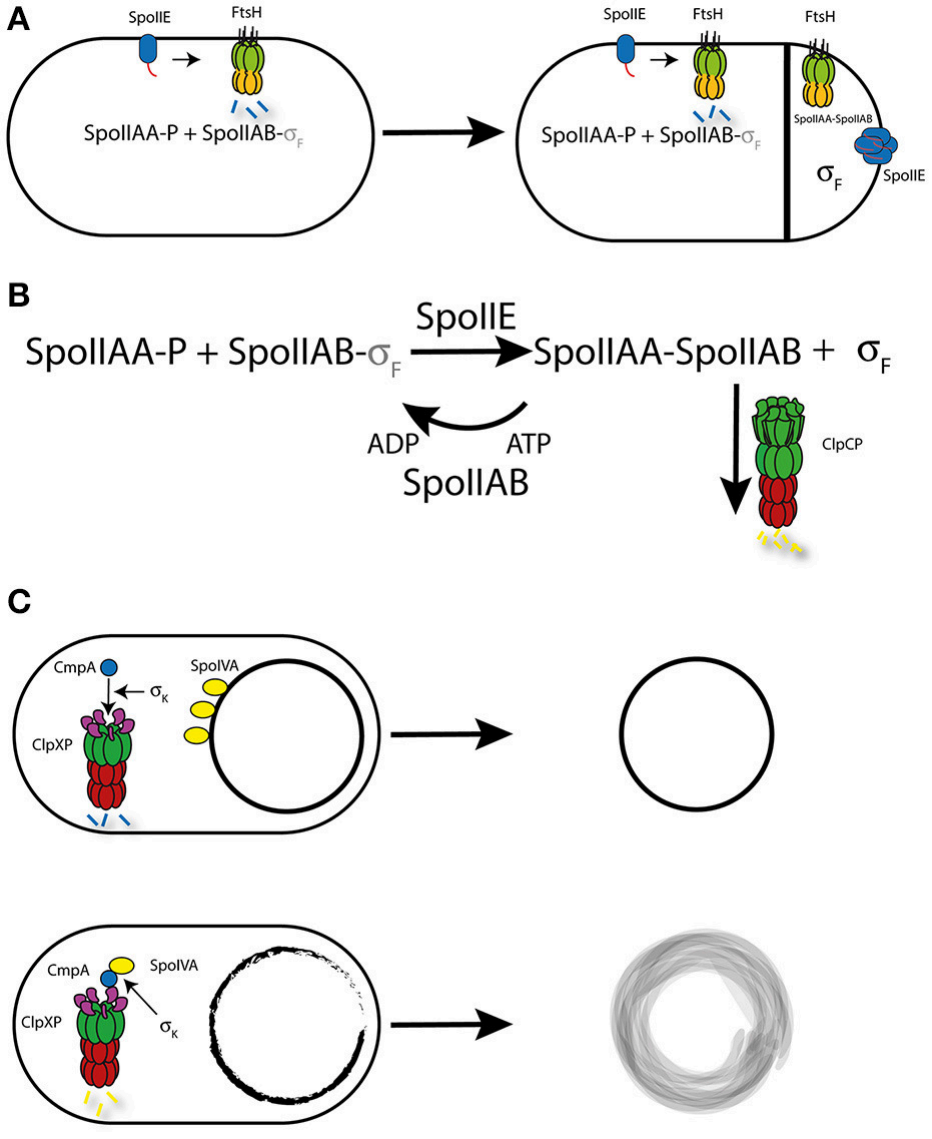
Regulation by Proteolysis during sporulation. **(A)** Model for the controlled degradation of SpoIIE by FtsH. In normal cells and the mother cell after asymetric division, monomeric SpoIIE accumulates at the divisome and is rapidly degraded by FtsH, who recognizes SpoIIE through a C-terminal Tag (red). This leads to the stabilization of phosphorylated SpoIIAA (AA-P) and in turn to the inactivation of Sigma F (s_F_) by SpoIIAB (AB). In the forespore, SpoIIE is enriched due to the close proximity to the division sites, which favors transfer of SpoIIE to the smaller forespore. The high concentration of SpoIIE promotes multimerization, in which the Tag-sequence is buried within the multimeric complex. This protects SpoIIE from FtsH-dependent proteolysis and leads to SpoIIE-dependent de-phosphorylation of SpoIIAA (AA), which in its unphosphorylated form can interact with SpoIIAB, thereby freeing and activating Sigma F, resulting in the cell-type specific activation of Sigma F. **(B)** Model for the control of Sigma F. The Kinase SpoIIAB (AB) is able to phosphorylate SpoIIAA (AA-P), which allows SpoIIAB to bind and inactivate Sigma F. Once the SpoIIE phosphatase (IIE) is activated, SpoIIAA becomes de-phosphorylated leading to the binding of SpoIIAB and the activation of Sigma F. To prevent further phosphorylation of SpoIIAA, SpoIIAB is targeted by ClpCP for degradation, which shifts the equilibrium toward unphosphorylated SpoIIAA. **(C)** Model for the CmpA-dependent control of spore integrity. In spores with a proper coat formation, CmpA is targeted by ClpXP and SpoIVA is stabilized, resulting in functional spore formation. In contrast, in cells with spores that display a defective coat, CmpA then mediates degradation of SpoIVA, which also depends on so far unknown factors controlled by Sigma K. This regulatory process results in cell lysis, preventing the spore development to proceed.

They showed that SpoIIE is subject to FtsH-dependent degradation in the mother cell, but is protected from proteolysis in the forespore. This specific stabilization results in the accumulation of active SpoIIE proteins in the forespore that lead to the forespore-specific activation of Sigma F (Figure 4A). The stabilization of SpoIIE in the forespore is not linked to differences in FtsH expression or activity in the different compartments.

Normally, SpoIIE is degraded by FtsH upon recognition of an N-terminal degradation tag. However, relocation of SpoIIE from the polar divisome to the cell pole results in stabilization of SpoIIE by a mechanism that is not yet fully understood but seems to involve SpoIIE oligomerization (Bradshaw and Losick, 2015; Figure 4A). Nonetheless, the local control of SpoIIE degradation is a great example of how proteolysis can be a crucial regulatory mechanism in the control of cell polarity.

Interestingly, FtsH is not the only AAA+ protease that is involved in the control of SigmaF activity. It has been shown that the ClpCP protease is responsible for the degradation of the anti-sigma factor SpoIIAB (Pan et al., 2001). Under normal growth conditions, SpoIIAB interacts and thus inactivates Sigma F (Duncan and Losick, 1993). This interaction is also thought to stabilize SpoIIAB. Upon de-phosphorylation of the anti-anti-sigma factor SpoIIAA by SpoIIE, SigmaF is liberated (Stragier and Losick, 1996) and SpoIIAB is subject to ClpCP-dependent degradation (Pan et al., 2001). Although, this proteolytic mechanism is not directly involved in the activation of SigmaF, it is required to maintain the stability of free Sigma F (Pan et al., 2001). Targeting of SpoIIAB for ClpCP-dependent degradation is enabled by the presence of the C-terminal amino acid sequence LCN (Pan et al., 2001; Pan and Losick, 2003). Interestingly, none of the described ClpC adaptors are involved in the proteolysis of SpoIIAB, which implicates a hitherto unidentified adaptor or molecular mechanism in this process (Kirstein et al., 2009b). Since artificially LCN-tagged proteins are also subject to degradation during exponential growth (Pan and Losick, 2003), it is unlikely that this process depends on a sporulation-specific adaptor protein (Figure 4B).

Regulated proteolysis is also involved in the control mechanisms ensuring proper spore formation. The ClpXP protease together with the adaptor protein CmpA are involved in the quality control of the spore envelope. In cells that produce spores with a proper spore envelope, CmpA is degraded through ClpXP-dependent proteolysis and sporulation continues. However, in cells that display defects in the spore envelope maturation, CmpA is stabilized and mediates ClpXP-dependent degradation of the coat morphogenetic protein SpoIVA. This proteolytic event causes instability and subsequent lysis of the spore, thereby ensuring that only properly assembled spores are produced within the population. The presence of ClpXP and CmpA is required but not sufficient for degradation of SpoIVA and also of CmpA itself. The proteolytic activity of this regulatory circuit depends on the presence of a specific signal or component that is under the control of the cell type-specific Sigma K. However, the nature of this signal or component is unclear and requires further investigation (Tan et al., 2015; Figure 4C).

The three mechanisms described above are examples of how regulated protein degradation is involved in the control of sporulation. In addition, evidence exists that AAA+ proteases and their associated proteolytic events play even more roles in the control of sporulation. A recent global high-throughput genetic screen highlighted the pleiotropic function of ClpC in the control of sporulation. Meeske and colleagues showed that cells lacking *clpC* had a dramatic defect in sporulation efficiency and displayed different phenotypes, such as delayed entry, asymmetric engulfment, reduced or no Sigma G activity and a concomitant small forespore phenotype (Meeske et al., 2016). This observation suggests that ClpC is specifically involved in the control of distinct but yet unknown regulatory events during sporulation.

### Motility and biofilm formation

A first analysis of *B. subtilis* strains with *clpC, clpX*, or *clpP* mutations suggested that these genes are important for swimming motility (Rashid et al., 1996; Liu and Zuber, 1998; Msadek et al., 1998). It was demonstrated that ClpCP and ClpXP enable motility via regulatory proteolysis of the transcription factors ComK, DegU and Spx, which directly or indirectly influence the transcription of flagellar genes (Liu and Zuber, 1998; Ogura and Tsukahara, 2010; Molière et al., 2016).

Interestingly, *B. subtilis* cells can switch from swimming to swarming motility on surfaces, which is accompanied by a hyperflagellation of the swarming cells (Kearns, 2010). The transcriptional activator SwrA determines the number of flagella in *B. subtilis* cells (Mukherjee and Kearns, 2014). This transition is controlled by regulated proteolysis of SwrA, which in swimming cells is targeted by the adaptor protein SmiA for LonA-dependent degradation (Mukherjee et al., 2015).

The transformation of *B. subtilis* cells from the motile to the sessile state depends on the presence of the SlrR regulatory protein. In the SlrR low state, motility and autolysis genes are expressed. In contrast, in the SlrR high state SlrR together with SinR repress motility and autolysis genes, resulting in long chains of sessile cell and biofilm formation. The induction of SlrR expression is well understood and depends on a complex three-protein regulatory circuit (Chai et al., 2010b; Norman et al., 2013). Interestingly, the switch from the SlrR high state to the motile, SlrR low depends on the controlled degradation of SlrR. It is not clear how SlrR is degraded, but it is known that an LexA-like auto-cleavage of SlrR is involved in SlrR stability. Interestingly, it was shown that the AAA+ protease ClpCP influences the stability of SlrR, but the precise molecular mechanisms have not yet been described (Chai et al., 2010a).

## Relevance of *B. subtilis* AAA+ protease complexes as a new target for antibiotics and for targeting virulence in gram-positive pathogens

Understanding the processes that determine stability and degradation of regulatory proteins under different environmental conditions in a model organism such as *B. subtilis* can provide important information that holds true for other bacterial species. AAA+ protease complexes mediate numerous essential aspects of bacterial physiology and are widely conserved among bacteria (Kirstein et al., 2009b; Sauer and Baker, 2011). They therefore represent promising targets for the development of novel antimicrobial therapies that are urgently needed to combat the rise in antibiotic resistance in pathogenic bacterial species (Raju et al., 2012; Culp and Wright, 2016). While it is estimated that up to 10% of pursued targets for drug development are proteases, therapeutics targeting bacterial proteolytic complexes are comparatively underrepresented (Drag and Salvesen, 2010).

AAA+ protease complexes are especially attractive as potential targets for novel antimicrobial therapies as they are essential for virulence in several pathogenic bacteria (Butler et al., 2006; Culp and Wright, 2016; Malik and Brötz-Oesterhelt, 2017). Because virulence is not generally essential for basic growth, the inhibition of virulence is believed to impose a lower evolutionary pressure on the pathogen. Therefore, AAA+ protease complex-targeted therapeutics might be less likely to induce resistance and might therefore represent a more durable anti-infective strategy (Rasko and Sperandio, 2010). Furthermore, adverse effects arising from modulation of the activity of human AAA-protease complex homologs are unlikely because of their low resemblance to the bacterial proteins (Raju et al., 2012). Another favorable feature of the large, multimeric AAA+ protease complex as potential targets for antimicrobials are the multitude of different activities and active sites that could be targeted by small molecules. Therefore, it is not surprising that AAA+ protease complex modulators—in contrast to well-established antibiotics—have substantially different mechanisms of action.

One class of AAA+ protease complex modulators, the acyldepsipeptides (ADEPs), was shown to exhibit an inhibitory effect on growth of several Gram-positive organisms, including *Staphylococci* and *Streptococci* by interacting with and dysregulating ClpP (Brötz-Oesterhelt et al., 2005). The molecular mechanism of ADEP activity was later investigated in more detail in a *B. subtilis* model, where it was shown that ADEPs influence ClpP activity in two ways. Firstly, they prevent ClpP from associating with its corresponding ATPase. This inhibits formation of the complete protease complex responsible for regulated proteolysis. Secondly, ADEPs enable ClpP to degrade unfolded proteins, making it independent from its ATPase and thereby deregulating substrate specificity (Kirstein et al., 2009a; Lee et al., 2010). It was later shown that ADEP4 kills *Staphylococcus aureus* persister cells by triggering indiscriminate, ClpP-mediated degradation of over 400 proteins (Conlon et al., 2013), including for example the cell division protein FtsZ (Sass et al., 2011). ClpP is not essential in *S. aureus*, but mutants lacking *clpP* were shown to be more susceptible to a range of other antibiotics. This suggests that ClpP reprogramming by ADEP4 in combination with other antibiotics may represent a possible strategy to eliminate persister cells (Conlon et al., 2013).

The working mechanism of ADEPs relies on both dysregulation of ClpP and disruption of the protease complex. Other natural compounds such as cyclomarin, ecumicin, and lassomycin, all of which bind to the N-terminal domain of the *Mycobacterium tuberculosis* chaperone ClpC1, were recently discovered. While the exact mode of action is still to be discovered, it was suggested that binding of the N-terminal domain of ClpC1 by ecumicin or lassomycin leads to inhibition of degradation of natural substrates, which would eventually lead to accumulation of proteins and toxicity (Gavrish et al., 2014; Gao et al., 2015; Culp and Wright, 2016). For cyclomarin, alteration of substrate specificity or structural changes that result in a more accessible axial pore of the protease complex were discussed. These hypotheses were based on the observation that the cyclomarin binding region at the N-terminal domain of ClpC1 overlaps with the site corresponding to the MecA interaction site on the NTD of *B. subtilis* ClpC (Schmitt et al., 2011; Vasudevan et al., 2013; Culp and Wright, 2016; Malik and Brötz-Oesterhelt, 2017).

Various questions regarding the mechanism behind antibacterial activity of these newly identified compounds targeting the NTD of AAA+ proteins remain unanswered (Culp and Wright, 2016; Malik and Brötz-Oesterhelt, 2017). Advancing the knowledge of AAA+ proteases in the *B. subtilis* model will help to understand how these promising targets for novel antimicrobial therapies against pathogenic bacteria work, but will also help to unravel the molecular mechanism of these antibiotics. In addition, understanding the molecular mechanism of the AAA+ protease complexes in *B. subtilis* help us to understand the mechanism of these molecular machines during virulence. AAA+ proteases contribute to virulence in two distinct ways. Firstly, they play a crucial role in removal of misfolded proteins that are formed under unfavorable environmental conditions. Secondly, proteases have been shown to contribute to virulence by controlling the abundance of regulatory proteins and transcription factors in response to diverse stimuli encountered during infection (Ingmer and Brøndsted, 2009). In Gram-negative organisms, several proteases of the AAA+ family contribute to virulence while in Gram-positive bacteria, the involvement of AAA+ protease complexes exceed the involvement of any other protease family (Ingmer and Brøndsted, 2009). In *Listeria monocytogenes* for example, ClpP was shown to regulate the expression of an essential virulence factor (Listeriolysin), the multiplication of the pathogen within macrophages, and the transcription of an actin-polymerizing protein (ActA) that is required for cell-to-cell spread (Gaillot et al., 2000). Additionally, the ClpCP-MecA complex was implicated in the downregulation of the surface virulence-associated protein, SvpA (Borezée et al., 2001). MecA was first described in *B. subtilis* as an adaptor protein for specific substrate recognition by ClpCP (Turgay et al., 1998). These examples support the notion that *B. subtilis* is a useful model organism for the study of the role of AAA+ protease complexes.

## Conclusion

The various AAA+ protease complexes of the Gram-positive model organism *B. subtilis* are involved in many cellular processes, ranging from protein homeostasis and protein quality control to stress response pathways and the control of cellular developmental processes. Adaptor proteins play an important role in substrate recognition during both general and regulatory proteolysis (Jenal and Hengge-Aronis, 2003; Kirstein et al., 2009b; Battesti and Gottesman, 2013; Joshi and Chien, 2016; Kuhlmann and Chien, 2017). More recently, a new protein modification mediated by the ClpC adaptor protein and protein arginine kinase McsB was discovered in *B. subtilis* (Fuhrmann et al., 2009). The possible role and function of this unusual protein modification (Mijakovic et al., 2016) is an area of active investigation (Elsholz et al., 2012; Fuhrmann et al., 2016; Trentini et al., 2016).

## Author contributions

All authors listed have made a substantial, direct and intellectual contribution to the work, and approved it for publication.

### Conflict of interest statement

The authors declare that the research was conducted in the absence of any commercial or financial relationships that could be construed as a potential conflict of interest.
